# Amorphous SiC Thin Films Deposited by Plasma-Enhanced Chemical Vapor Deposition for Passivation in Biomedical Devices

**DOI:** 10.3390/ma17051135

**Published:** 2024-02-29

**Authors:** Scott Greenhorn, Edwige Bano, Valérie Stambouli, Konstantinos Zekentes

**Affiliations:** 1 The Institute of Electronic Structure and Laser of the Foundation for Research and Technology-Hellas (MRG-IESL/FORTH), GR-70013 Heraklion, Greece; 2 Laboratoire des Matériaux et de la Génie Physique, Université Grenoble Alpes, Centre National de la Recherche Scientifique, Institut Polytechnique de Grenoble, 38016 Grenoble, France; 3 Centre de Radiofréquences, Optique et Micro-nanoélectronique des Alpes, Université Grenoble Alpes, Centre National de la Recherche Scientifique, Institut Polytechnique de Grenoble, 38016 Grenoble, France

**Keywords:** a-SiC, amorphous, PECVD, biomedical, passivation

## Abstract

Amorphous silicon carbide (a-SiC) is a wide-bandgap semiconductor with high robustness and biocompatibility, making it a promising material for applications in biomedical device passivation. a-SiC thin film deposition has been a subject of research for several decades with a variety of approaches investigated to achieve optimal properties for multiple applications, with an emphasis on properties relevant to biomedical devices in the past decade. This review summarizes the results of many optimization studies, identifying strategies that have been used to achieve desirable film properties and discussing the proposed physical interpretations. In addition, divergent results from studies are contrasted, with attempts to reconcile the results, while areas of uncertainty are highlighted.

## 1. Introduction

Amorphous silicon carbide (a-SiC or in hydrogenated form, a-SiC:H) is a promising material for various applications in opto- and micro-electronic devices. This thermally and chemically stable wide-bandgap dielectric material has superior mechanical strength, high radiation resistance, and high optical transmittance [1]. Amorphous SiC appears to be suitable as protective coating against corrosion, moisture, etching, and abrasion [2,3], and has shown promise in optoelectronics [4,5], electronic device insulation [6,7], as well as for biomedical applications [8,9,10,11,12,13,14,15,16,17]. Amorphous SiC is specifically considered as an advantageous material for the isolation of neural implantable devices [11,18,19,20] as it is inherently insulating; has high-K dielectric properties, high wear resistance, and direct binding to Si; and works well as a durable, biocompatible, and hemocompatible coating. Each application has a tendency to focus on different a-SiC composition ranges, where optoelectronic applications prefer more conductive Si-rich films, and electronic device insulation tends to optimize for highly resistive C- and H-rich films, while mechanical and biomedical applications aim for stoichiometric, robust, and chemically inert films.

The passivation layer of an implantable device [21,22] is responsible for isolating adjacent channels electrically, while also protecting the device from the immune response after implantation [23]. In many cases, the thickness of the passivation layer is also non-negligible (up to several microns [24]), and thus its mechanical properties (stress, Young’s modulus, hardness) have a significant effect on the device behavior as well [25]. An optimized passivation layer displays high resistivity, high chemical resistance, low stress, excellent biocompatibility, and negligible toxicity [26]. The mechanical properties must strike a balance between minimizing mechanical mismatch with the brain [27], ensuring high flexibility to minimize micromovement-induced issues [28], and ensuring sufficient rigidity and strength for precise insertion into the brain [29,30].

a-SiC compares favorably to typical passivation materials, particularly in terms of the robustness and lifetime. In particular, the dissolution rate in phosphate-buffered saline (PBS) is an order of magnitude slower than that of more conventional passivation materials like Si_3_N_4_ or SiO_2_ [18,31,32,33], and significantly longer than parylene or polyimide passivation [34,35] due to a-SiC’s reduced permeability to water. This results in longer implant lifetimes and reduces material leeching into surrounding tissue. Implants coated in a-SiC showed reduced etching in a buffer solution compared to fluorapatite (dental glass) ceramic, particular for low pH solutions [17]. While the rigidity of a-SiC makes matching the mechanical properties of tissue more difficult [36,37], its strength allows for very thin devices (~6 µm) and thus sufficiently flexible behavior to minimize the biological response and at the same time reduce insertion trauma [30,38,39].

a-SiC was first used in biomedical applications as a biocompatible coating for stainless steel stents starting in 1996 [11,14,40], where it demonstrated significantly reduced protein activation compared to the uncoated implants. Further studies have cemented a-SiC as a biocompatible and hemocompatible material for a variety of applications including bone implant scaffolds and biosensors (2003-present) [12,18,41,42,43,44,45]. In particular, a-SiC has been used as a passivation material for SiC [24,46,47], polymeric [38], and metallic [39,48] implantable microelectrode arrays, starting in 2003, as well as a coating for prefabricated Utah arrays [18,19], showing its potential for implantable electrical devices. Note that the US-FDA (Food and Drug Administration) approved in 2005 under PMA #P030037 an intravascular stent coated with a-SiC. Finally, a-SiC has also been used as the structural support material as well as passivation for implantable electrodes [39,48].

A previous general review of a-SiC is given by Bullot and Schmidt [49], reviewing results in a-SiC based on data published between 1968 and 1987. Another review on a-SiC film developments was presented by Choi [50], based on publications between 1987 and 2000. Fraga et al. [51] provide a more recent synthesis of results across a wide variety of deposition methods, but do not cover film optimization for any deposition method. A review of a-SiC biocompatibility and suitability for biomedical applications was performed by Mahmoodi and Ghazanfari [52] in 2011 without including details on the deposition and physical characterization of the material. Similar topics have also been addressed in sections of a chapter by Locke et al. [33] in 2012 and an article by Saddow [10] in 2022, both of which focus on crystalline SiC material more generally and only briefly discussing the a-SiC material aspects. a-SiC is also briefly addressed in a more general review by Kaloyeros and Arkles [53] in 2023. While their review provides a comprehensive look at deposition methods and applications of SiC, it does not investigate in detail the relation between process conditions and material properties.

With ongoing research and improved deposition processes [6,7,31,54,55,56,57,58] as well as increasing opportunities for applications of a-SiC, there is now a need for a comprehensive introduction to a-SiC film optimization. To maintain a reasonable scope for the review, the main focus will be on PECVD deposition optimization as it has the most extensive literature and is most frequently used in studies on biomedical applications. The result is a synthesis of the current state of knowledge of the optimization of PECVD a-SiC, providing a starting point for designing optimization studies of a-SiC to new researchers, outlining demonstrated processes to obtain films with desirable properties for various biomedical applications, and reviewing recent results for those already working in a-SiC technology. Our proper results are incorporated in many cases to strengthen our conclusions on a-SiC optimization.

## 2. Deposition of a-SiC Films

A survey of papers on a-SiC films published over the past 30 years shows that the majority of such films were deposited by PECVD, and the second-most common technique is sputtering.

### 2.1. Sputtering Method Employed for a-SiC Film Deposition

A summary of deposition techniques given in [59] describes sputtered SiC films as having “fair” uniformity, “very good” substrate versatility, “good” stress control, and “fair” throughput. The same study also reports that, due to the chamber geometry with the sputtering target far from the substrate, the sidewall coverage is often poor, which results in poor conformal coatings for non-planar devices.

a-SiC films can be deposited via multiple sputtering procedures, including a single SiC target, a single target (Si) in a C-containing atmosphere, or co-sputtering with a Si target and a C target [49,60]. A SiC target is preferred as the composition of the sputtered film usually replicates that of the target, allowing great control of the resulting film’s composition. Unhydrogenated films are usually prepared by sputtering of a polycrystalline SiC target using argon ions (Ar^+^) [49]. This also leads to a very low level of impurities in the resulting sputtered film. As the sputtering process removes Si–C molecules rather than individual C or Si atoms, the method results in perfect stoichiometry for the deposited film. These films exhibit higher chemical resistance due to the lack of H bonds.

It is noted in one study that sputtered a-SiC films deposited at room temperature have been observed to display high pinhole density and low resistivity [60], which is said to make them unsuitable for biomedical applications. However, optimized resistivity values for sputtered films (often deposited at temperatures above 150 °C) range from 10^4^ Ω·cm [61] up to 10^11^ Ω·cm [62] for non-hydrogenated sputtered films and as high as 10^14^ Ω·cm for hydrogenated films [63], and do not report the presence of pinholes. Stress values range from highly compressive (−1400 Mpa) to slightly tensile (100 MPa) depending on the deposition conditions [64].

The optical constants (refractive index, extinction coefficient, bandgap) of a-SiC films obtained by sputtering have been reported to show a variability of up to 10%, including by ageing or annealing, that is not explained purely by the deposition process or film composition [65]. This suggests that as-deposited sputtered a-SiC films do not result in time-stable films, a factor which must be considered when determining deposition recipes.

### 2.2. PECVD Method Employed for a-SiC Film Deposition

PECVD a-SiC films have consistently been shown to have the best electrical and mechanical properties of the various growth methods [60]. The previously-referenced summary of deposition techniques [59] describes PECVD as having “fair” uniformity, “very good” substrate versatility, “very poor” stress control, and “very good” throughput. In particular, PECVD has been shown to be vulnerable to “edge effects” in small samples, with non-uniform edges at the extreme edges of the samples [66]. This can be reduced by moving to Induction-Coupled Plasma (ICP) CVD [67], although the literature available for this method is substantially smaller than for conventional PECVD.

PECVD [68] is a variation on CVD techniques using gas-phase precursors in an inert carrier gas to grow thin films, which allows low-temperature growth due to capacitive coupling with a plasma. Ionizing the gases in the chamber results in free electrons, which transfer energy very efficiently to the gas while the neutral atoms remain near ambient temperature. The energetic electrons can induce high-energy processes (precursor dissociation, creation of free radicals) desired for film deposition without the drawbacks of high-temperature deposition for the substrate (crystallization, damage to heat-sensitive substrates, etc.).

The plasma tends to be more positively charged than the chamber (due to the high mobility of electrons); in other words, there is a consistent flow of electrons from the plasma to the chamber walls and substrate. All these surfaces receive an energetic ion bombardment due to the induced electrostatic force. This bombardment results in a high film density and few contaminants, but has also been noted to increase film stress [6,69]. With high-density plasmas, sputtering can occur simultaneously, which planarizes the film by filling in cavities.

PECVD of SiC can be performed using a single gas precursor providing the silicon and carbon atoms or can be performed by using separate precursors for the silicon and the carbon atoms individually. Unlike sputtering, however, the preferred method here is to use separate precursors, primarily using a mixture of silane and methane (SiH_4_ + CH_4_) gases. Precursors of ethylene (C_2_H_4_) [70,71,72] and methyltrichlorosilane (CH_3_SiCl_3_) [73] have also been reported. Inert (such as Ar or He) and active (such as H_2_) carrier gases are also frequently used.

In addition to the precursor gases and their flow rates, the chamber temperature and pressure as well as the radio frequency (RF) power and, occasionally, frequency can be independently controlled in most PECVD setups. Each of these parameters has been reported to have distinct influences on deposited film properties, which will be discussed in Section 3.

As PECVD chambers can be used to deposit multiple materials, it is necessary to ensure cleanliness prior to a-SiC deposition. For proper recipe repeatability, it is crucial to guarantee that start conditions of the chamber are always the same. Many chemistries are used to clean the chamber and prevent deposited materials from building up on the chamber surfaces. Most studies [60,74] agree that a plasma cleaning pre-treatment using fluorinated (CF_4_, NF_3_, …) and oxygen-containing (O_2_, N_2_O) gases is useful to remove a buildup of material in the chamber. In addition, a short pre-deposition of the material before introducing the sample can be performed to ensure consistent chamber conditions throughout the full deposition process. A flush [60] or sputter cleaning of the sample [75] with Ar prior to deposition has been reported as necessary to avoid pinhole formation. The chamber is evacuated to pressures below 5·10^−6^ Torr just prior to process gases starting to flow. 

### 2.3. Optimal Deposition Technique for Biomedical Applications

While sputtering can achieve films with some desirable properties, it has not found use in biomedical applications, which prefer PECVD [8,9,11,12,13,14,15,16,17,18,20,24]. This is mainly due to the relatively poor sidewall coverage and resulting inhomogeneous coating achieved by sputtering. As such, this review will focus primarily on the physics and optimization of PECVD a-SiC in order to obtain desirable film qualities for these applications.

A potential application for sputtered a-SiC is in multilayer, multifunctional a-SiC films [24]. In the referenced study, sputtered a-SiC was used as a thin chemical protection layer on top of a thicker electrical passivation layer of a-SiC:H deposited by PECVD. This approach allows the combination of the high electrical isolation from PECVD (see next section) in addition to the maximal chemical passivation from stoichiometric sputtered a-SiC.

## 3. Controlling a-SiC Film Properties for PECVD

Most studies of a-SiC film deposition focus on a subset of the film properties and deposition parameters, studying individual interactions between single deposition variables and properties of the resulting films. The most commonly studied deposition parameters include the flow rates of precursor gases, the inclusion of H_2_ as a precursor, the deposition temperature, the RF power, and the pressure of the chamber. Post-treatment of the films involving plasma and annealing has also been studied. Various properties are studied to characterize the resulting films: the composition, in terms of Si, C, H, and occasionally O; the bonding structure in terms of Si–C, Si–H, and C–H, as well as Si–Si and C–C; the film organization in terms of an average number of bonds per atom, the surface roughness, the conductivity or resistivity of the films, the optical properties including the bandgap and refractive index, the stress, the hardness, the Young’s modulus, and the resistance to a chemical attack. A thorough analysis of the relationship between the deposition conditions and the a-SiC film properties is performed below and a conclusion on optimal deposition conditions for a-SiC for biomedical applications is reported for each material property.

### 3.1. Stoichiometry of a-SiC Films

The stoichiometry of a-SiC films can be varied from pure Si to pure C. If a layer is not stoichiometric, the following symbolism of a-Si_x_C_1−x_ is employed.

In the case of an amorphous binary material such as silicon carbide (a-Si_x_C_1−x_), three main types of bonding are possible: Si–C (heteronuclear), Si–Si, and C–C (homonuclear). Most of the free energy of the amorphous phase is stored in the material as angular distortion in the Si–C bonds [76], which is always present in the a-SiC. Moreover, other types of defects such as chemical disorder (i.e., the presence of homonuclear Si–Si and C–C bonds) and dangling bonds are present, as well as bonds to typical ambient atoms such as H or O, which are present in most films.

For stoichiometric films with 50% Si and 50% C and with perfect chemical ordering, a given Si is expected to be surrounded by four C, and vice versa. As the composition ratio *x* is varied, one would expect that the relative proportions of the three types of bonds also change (chemical disorder). Since C–C bonds are thermodynamically more stable than Si–Si and Si–C bonds, it is probable to find C–C bonds already in a material with a C/Si fraction slightly higher than 1, starting around C/Si = 1.1 and increasing with the C content [76]. At higher carbon concentrations, clusters may be formed, which are expected to contain graphitic configurations. Annealing a-SiC films in a dry atmosphere is also reported to increase graphitic cluster formation [77]. Also, the consistent Si and C lattice is in most cases broken by the incorporation of hydrogen atoms, which only bond to a single Si or C atom. The organization of the films can be given in terms of an <r> value describing the average number of bonds for a given atom in the network [56,78].

a-SiC film stoichiometry has been shown to affect the conductivity (Section 3.2.1), optical bandgap (Section 3.3.1), refractive index and dielectric constant (Section 3.3.2), stress (Section 3.4.1), and hardness and Young’s modulus (Section 3.5.1).

#### 3.1.1. Variation of Stoichiometry with Precursor Gas Ratio

**Figure 1:**
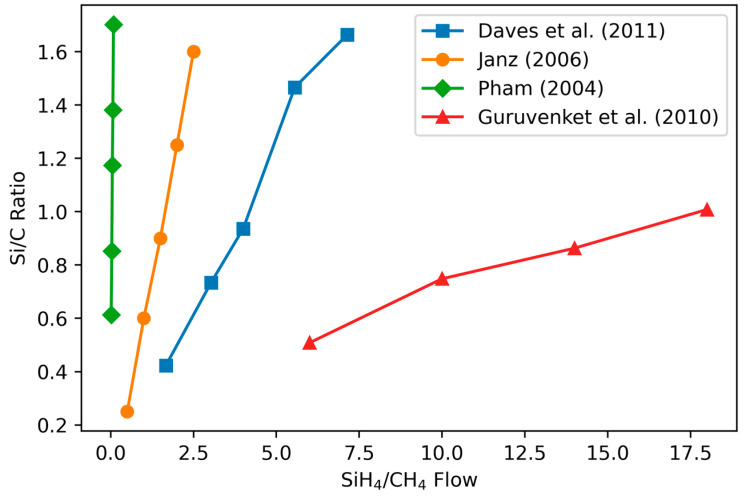
Comparison of measured Si/C atomic ratios to precursor SiH_4_/CH_4_ ratios for all reproducible studies. Compositional studies were performed using X-ray Photoelectron Spectroscopy (XPS) (Daves et al.), Elastic Recoil Detection (Time-of-Flight mode) (ERD-ToF) (Guruvenket et al.), electron-probe microanalyzer (EPMA) (Pham), and Auger Electron Spectroscopy (AES) (Janz et al.). Daves et al. used 1000 mTorr pressure and high temperature (400 °C) with a SiH_4_/CH_4_ ratio of 0.25; Janz used a temperature of 350 °C, pressure of 37.5 mTorr, and power density of 250 mW/cm^2^; Pham used a temperature of 400 °C, 2250 mTorr pressure, and a power of 100 W; and Guruvenket et al. used temperature of 300 °C and pressure of 100 mTorr. Data from [55,79,80,81]. Reproduced with permission.

Importantly, the Si/C ratio in the film does not match the ratio of silicon to carbon in the precursor gas flows as SiH_4_ is more reactive than CH_4_ (and indeed several other C-containing precursors such as C_2_H_4_) [79]. Studies show that for a given deposition setup, the relation between the precursor gases’ ratio and film composition is nearly linear, with different slopes for each experiment (Figure 1) [55,79,80,81].

**Figure 2:**
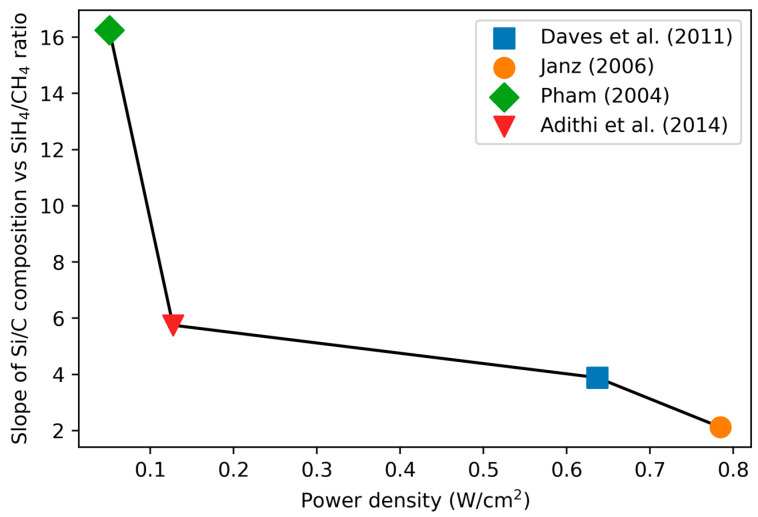
Comparison of the slope of the Si/C curve (Figure 1, right) to the power density for the studies, where available. Values calculated from [55,79,80,82].

The slope of the composition–precursor ratio curve correlates most strongly to the power density used (power/chamber area, Table 1, Figure 2), with high power densities resulting in a less steeply increasing atomic Si/C composition as a function of the precursors (see next section on RF power). The chamber size used for the calculation is approximate, based on the reported maximum wafer size for the PECVD system used.

In conclusion, the composition of a-SiC films is linear as a function of the ratio of SiH_4_ and CH_4_ precursors, with the slope determined by the other deposition characteristics, in particular, the power density.

#### 3.1.2. Variation of Stoichiometry with RF Power

**Figure 3:**
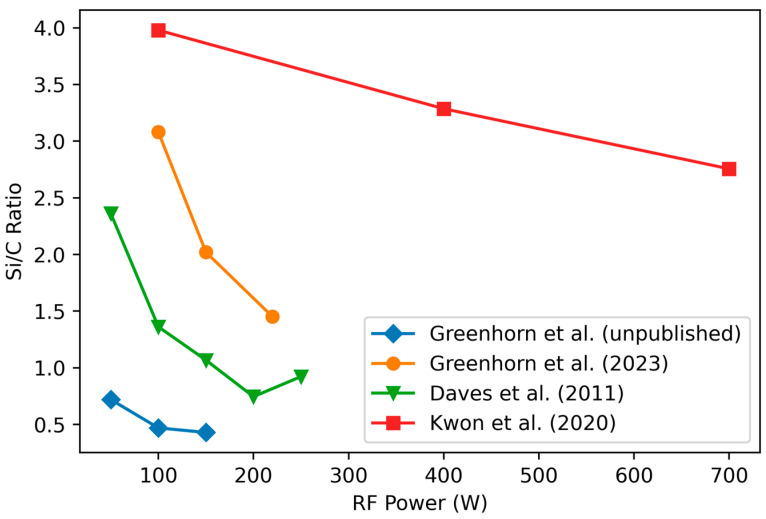
Change in Si/C ratio as a function of RF power. The studies by Greenhorn et al. use C_2_H_4_ as a C precursor, and the unpublished data use much lower precursor flow rates combined with high Ar flow. Daves et al. used 1000 mTorr pressure and high temperature (400 °C) with a SiH_4_/CH_4_ ratio of 0.25 while Kwon used a large industrial-type PECVD chamber with high flow rates, a pressure of 2500 mTorr, and a very high temperature of 550 °C and precursor gases of Si_2_H_6_ and CH_4_. Data from [55,84,85] and additional unpublished data from the authors. Reproduced with permission.

Three studies [55,84,85] have found that increasing the RF power decreases the Si/C ratio of the film (Figure 3). This can be explained by the relative difficulty of forming reactive species from CH_4_ compared to SiH_4,_ due to the higher bond energy of C–H (98.8 Kcal/mole) compared to that of Si–H (70.9 Kcal/mole). Higher RF powers allow reactive species of CH_4_ to be formed and independently adsorbed/bonded on the substrate [54]. Once C-reactive species are on the growing surface, they form stable C–H much easier than Si–H bonds (see section below on hydrogen incorporation). At low RF powers, mainly reactive species of SiH_4_ are formed, and C is mostly incorporated through whole CH_4_ molecules bonding to the SiH_4_ radicals present on the surface [86,87,88]. This situation with the presence of SiH_4_ radicals but not CH_4_ radicals is referred to as a “low-power-density regime” with the most commonly-used case of a “silane-starving plasma” where all silane molecules entering the chamber are radicalized [19,54]. Silane-starving plasma requires both an RF power sufficiently low to radicalize only silane as well as a sufficiently low silane flow such that all the silane can be radicalized. Silane-starving plasma has been reported using either very low power density (8.3 mW/cm^2^) with an SiH4/CH4 ratio near 0.3 [54], or using a higher power density (250 mW/cm^2^) combined with a lower SiH4/CH4 ratio of 0.17 [19]. This has been reported to result in films with diamond-like properties and high Si–C ordering [86].

Note that one study by Deku et al. [89] does not report a change in the composition as a function of the RF power.

In conclusion, most studies agree that a-SiC films become more Si-rich as the RF power is decreased.

#### 3.1.3. Variation of Stoichiometry with Deposition Temperature

The deposition temperatures used for a-SiC typically range from 150 °C up to 450 °C. Diaz-Botia et al. [60] note more C–H bonding and less Si–C bonding for films deposited at 200 °C than for those deposited at 350 °C using precursor SiH_4_/CH_4_ ratios from 0.1 to 0.4. Hsu et al. [19] also found that films deposited at 200 °C have less Si–C compared to films deposited at 400 °C. No variation in the Si/C or O content ratio has been observed [19,60,90]. 

However, Huran et al. [91] found an increase in the Si/C ratio (from 0.625 to 0.857) as the temperature increases from 250 °C to 450 °C. Deku et al. [89] also found a slight increase in the Si/C ratio for deposition temperatures above 300 °C, for films near Si/C = 0.5.

In conclusion, studies do not agree on the influence of deposition temperature on Si/C composition, with some reporting a slight increase in Si with increasing temperature while others report no effect. Nevertheless, most studies agree that the increase in chamber temperature increases the Si–C bonding. 

#### 3.1.4. Presence of Hydrogen in a-SiC

PECVD a-SiC films contain hydrogen in a sufficiently important quantity to change the stoichiometry and in this case the film is often referred to as hydrogenated and denoted as a-Si_x_C_1−x_:H_y_. Reported hydrogen contents range from 15 to 58% [56,92,93,94,95,96] for films deposited using SiH_4_–CH_4_ gas mixtures, Ar as carrier gas, and no hydrogen flow. Hydrogen acts as a terminating atom in the crystalline structure, being 1-fold coordinated compared to Si and C, which are 4-fold coordinated. The presence of hydrogen thus disrupts the regular structure of Si and C and functions as a terminating atom.

In principle, hydrogen incorporation is unavoidable due to the PECVD gas precursors containing hydrogen (CH_4_, C_2_H_4_, etc.). C–H bonds are more stable than the Si–H bonds, so carbon atoms are expected to be more hydrogenated than silicon atoms and thus C–H bonds are more prevalent than Si–H bonds. Demichelis et al. [92] found that hydrogen content in the film increases linearly with the amount of CH_4_ in the precursor gas, which supports the result that hydrogen incorporation in the film is brought on primarily by CH_4_. This is also found by King et al. [56] and by Fujimoto et al. [71].

Using H_2_ as the main carrier gas has been shown to decrease CH_n_ and SiH_2_ groups while increasing Si–C bonding [19,60,76]. In [19], the effect is reported to be magnified in the silane-starving case. The same study identifies two potential mechanisms: either that H flow removes methyl radicals as methane before they can be deposited, or that hydrogen plasma preferentially etches CH groups, removing H from the deposited material. The structural result is a decrease in microvoids (and increase in film density) and a reduction in graphitic cluster formation [56,97].

Decreasing the RF power is found to increase the H content of the films [71,84,91]. King et al. [56] also notes that very-low-frequency RF (300–400 kHz) has been used to achieve high H content (>50%) in some films. Yang et al. [90], Lee et al. [70], and Herrera-Celis et al. [54] observed that hydrogen content decreases with increasing deposition temperature.

In conclusion, hydrogen incorporation always occurs in PECVD a-SiC with hydrogen-containing precursors. C–H bonds are more stable than Si–H bonds, so most H is incorporated with the carbon-containing precursor [49], and correspondingly, the hydrogen content tends to increase for higher C content [92]. The H content can be reduced by increasing the temperature [54,70,90] or RF power [56,71,84,91], or by using H_2_ as a carrier gas [19].

#### 3.1.5. Presence of Oxygen in a-SiC

Oxygen incorporation is less studied than hydrogen in a-SiC films but is mentioned in several studies. In particular, oxygen content ranging from 0.1 to 23 atomic % is reported [6,20,55,74,98]. The study with the highest reported O content (23%) notes that this was due to chamber contamination [20]. Another study [89] examined more samples in order to observe trends in oxygen content, finding increased oxygen in the bulk in films deposited at lower temperatures (150 °C, 250 °C vs. 325 °C, 350 °C). This is consistent with the expectation of increased porosity for low-temperature films described in Section 3.1.3, which allows O to percolate through the ‘porous maze’ [99]. XPS measurements allowed the binding structure of the incorporated oxygen to be studied in more detail. For high temperatures, the O atom presence is well-described by O–Si–C bonds, while the larger and broader peak for lower temperatures suggests both O–Si–C and O–Si bonding. The O–Si–C peak was observed to increase over time with the sample exposed to the air. Yang [90] found an increase in C–O–H bonds in films with very low C/Si precursor ratios.

Bulk measurements report significantly lower O content compared to the surface due to the formation of the surface oxide [70]. A surface oxide is also reported by Tomastik et al. [100] for doped a-SiC, where the oxide layer increases when the sample is annealed at higher temperatures. The oxide has a thickness of approximately 70 nm and is reported to be mechanically softer than the bulk a-SiC.

#### 3.1.6. Stoichiometry of a-SiC Films for Biomedical Applications

Most demonstrations of implantable devices employ stoichiometric or near-stoichiometric a-SiC films when optimizing for biomedical applications [18,19,44]. Coatings for bone implants have used slightly more C-rich films (Si/C = 0.745) [15]. Stoichiometric composition is associated with high Si–C bonding, which results in robust, long-lasting films. For the same purpose, the H_2_ content must be minimized, which can be achieved by using silane-starving plasma and adding H_2_ flow [19,54]. These deposition conditions allow a wider range of compositions to be explored in order to optimize other material properties.

### 3.2. Resistivity of a-SiC Films

a-SiC resistivity has been measured using both in-plane [101,102] and vertical (using metal-insulator–metal capacitor-like structures) [18] measurements, and indirectly through leakage current measurements (see Section 3.6.1). The two main methods have not been compared with a-SiC deposited using the same parameters. Unlike single-crystal SiC, the high resistivity of a-SiC films [6,76,102,103,104,105] comes about from the amorphous microstructure and relatively low deposition temperatures used compared to crystalline SiC. The lower deposition temperature prevents the activation of dopants from the deposition, thereby resulting in an electrical insulator. The mechanisms of electrical conduction in a-SiC films are not trivial [106,107,108,109] and are not pursued in detail here in favor of focusing on the methods of controlling the resistivity during deposition. The conductivity of a-SiC is known to increase in a power relationship as the measurement’s AC frequency is increased for frequencies up to 1 MHz [96,109], resulting in resistivity decreases of up to 3 orders of magnitude from DC to 1 MHz. This reduces the passivation capability of a-SiC for high-frequency applications.

#### 3.2.1. Variation of Resistivity with Stoichiometry

**Figure 4:**
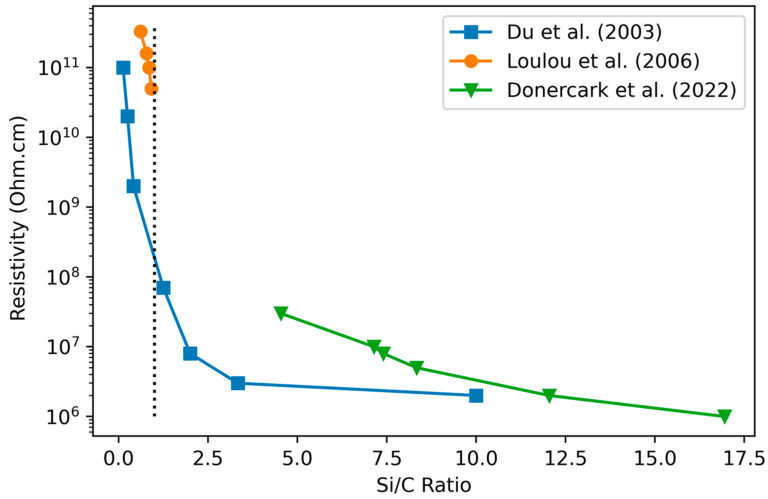
Resistivity as a function of precursor ratio. Stoichiometric (1:1) composition is marked with a vertical dotted black line. Du et al. used C_2_H_4_ as a precursor gas with H_2_ as a carrier, while Loulou et al. used CH_4_ and He carrier. Donercark et al. used SiH_4_, CH_4_, and H_2_ gases. From [58,102,110]. Reproduced with permission.

According to Du et al. [110], the resistivity decreases exponentially from 1·10^11^ to 1·10^6^ Ω·cm as the atomic Si/C ratio increases, showing that a higher fraction of C leads to higher resistivity. This is explained in terms of nanocrystallinity, as crystalline Si regions reduce the resistivity. High Si content is also associated with 4-coordinate sp^3^-like σ bonding, which does not allow complete relaxation and thus the elastic strain is relieved by dangling bonds, which contribute to conduction [109]. Increasing the number of C atoms breaks the Si crystalline pattern, forming C–C diamond-like bonds [105], and thus increasing the resistivity. Loulou et al.’s [102] and Donercark et al.’s [58] results are consistent with Du et al. [110] across their respective ranges (see Figure 4).

Desalvo [76] found that H incorporation in PECVD-grown a-SiC:H does not influence the dark conductivity. Alternatively, King [6] found very high resistivities for highly porous, H-rich films, finding resistivities above 10^16^ Ohm.cm for films with H% greater than 40%. However, this study increased both H and C content simultaneously, making the influence of H alone uncertain. Dutta et al. [111] compared sputtered hydrogenated and unhydrogenated a-SiC, and found a significant increase in resistivity from a-SiC to a-SiC:H along with smaller increases as the partial pressure of H_2_ was increased.

In conclusion, resistivity increases with the amount of carbon in the film according to studies using PECVD deposition. Hydrogen content may also play a role by increasing the nanoporosity but is difficult to distinguish from the variation of C content in the case of PECVD. Si-rich films, by contrast, are reported to have regions of nanocrystalline Si, which cause a lower resistivity. The combination of the two effects describes the reported results well: as Si content decreases, nanocrystalline Si decreases and nanoporous C–H increases, resulting overall in an increase in resistivity.

#### 3.2.2. Variation of Resistivity with Deposition Temperature

**Figure 5:**
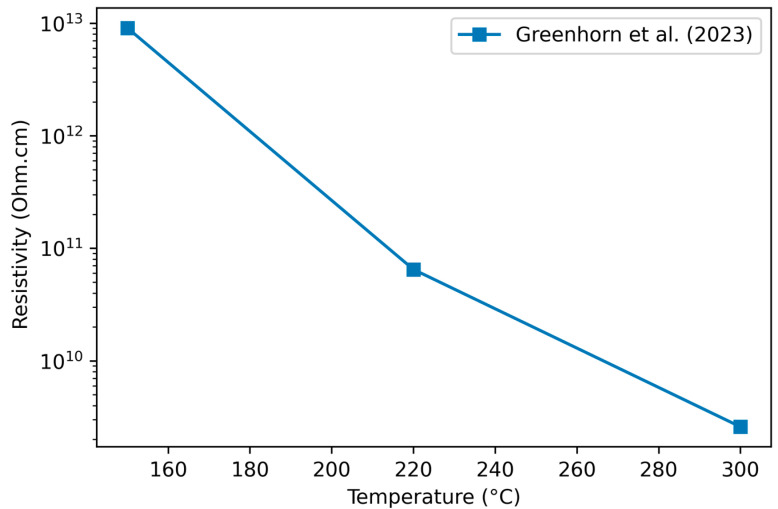
Variation of resistivity with deposition temperature. The films were deposited at 300 °C with 220 W RF power and 1200 mTorr pressure with a SiH_4_/C_2_H_4_ ratio of 0.57. From [84] and reproduced with permission.

One study [84] reports a resistivity dependence on deposition temperature under consistent conditions; lower temperatures correspond to higher resistivity (Figure 5). Du et al. [110] suggest that higher temperatures are more likely to form nanocrystalline Si regions in the film, which exhibit increased conductivity compared to a-SiC with more Si–C bonding. Harder et al. [11], alternately, attribute the increase in conductivity to the lower quantity of hydrogen in films deposited at high temperature, which increases the density of states in the bandgap.

Prolonged studies of electrical isolation over time find that the use of lower deposition temperatures (200 °C vs. 350 °C) gives higher resistivity when measured immediately, but exhibit a higher rate of eventual failure in thin films due to leakage currents through micropores in the film [60]. This is consistent with the model of porous material, leading to high initial resistivity but also allowing chemical attacks to easily form channels. 

#### 3.2.3. Varying a-SiC Resistivity by Doping and/or Post-Deposition Annealing

Studies in optoelectronics have sought to achieve lower-resistivity a-SiC by doping. Meanwhile, amorphous materials are often relatively difficult to dope, as the large existing variations in local structure accommodate impurities without strongly affecting the electronic properties. However, studies have shown that a combination of doping and annealing can result in an appreciable decrease in resistivity.

Common dopants for a-SiC films are nitrogen (from NH_3_) [103], phosphorus (from PH_3_), or boron (from B_2_H_6_) [74,112]. By adding a flow of a dopant precursor with proportions of up to 10% of the combined silane and methane precursor flow rates, resistivity decreases by approximately two orders of magnitude. An additional annealing step, at 650 °C for two hours, was reported to further lower the resistivity by an additional four orders of magnitude [74]. 

Vetter et al. [101] further studied the effect of low-temperature annealing in the case of low-RF-power films deposited at 400 °C. Transitions in the resistivity are observed at T = 80 °C, where a decrease in resistivity is observed, and at T = 170 °C, where a permanent increase in resistivity is observed. The first decrease is attributed to the breaking of weak Si–H and C–H bonds, which are expected to be plentiful due to the low RF power used during deposition, leaving dangling bonds that result in higher conductivity. The permanent increase in resistivity is then attributed to the reformation of covalent bonds in the a-SiC network, reorganizing to reduce dangling bonds in a more stable arrangement that is not destroyed by further heating/cooling. High conductivity has been achieved in a-SiC using a combination of doping and annealing [113].

#### 3.2.4. Resistivity of a-SiC Films for Biomedical Applications

High-resistivity a-SiC is desirable for the passivation of neural implants, with values of the order of 10^12^–10^13^ Ω·cm reported in [11,18] for near-stoichiometric films. Although increasing the C content could further increase the resistivity, this typically results in a decrease in chemical inertness (see Section 3.6.1). Therefore, near-stochiometric films are employed for this class of biomedical applications where increasing the C content is undesirable.

### 3.3. Optoelectronic Properties of a-SiC Films

#### 3.3.1. Optical Bandgap

The Urbach rule [114]—in which optical absorption at the band edge is found to be exponential with respect to the photon energy—is observed in an extraordinarily large number of materials systems. This rule is used for the determination of the optical bandgap in amorphous semiconductors including a-SiC. Various measured values have been reported for the a-SiC optical bandgap, showing the dependence of this parameter with growth conditions.

**Figure 6:**
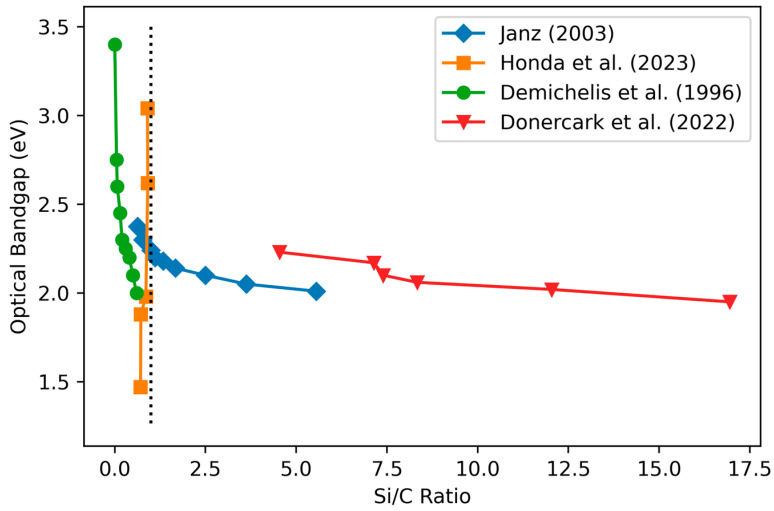
Bandgap dependence on C/Si stoichiometric ratio. A dotted black line marks stoichiometric composition. Janz used SiH_4_ and CH_4_ gases, a temperature of 350 °C, pressure of 37.5 mTorr, and power density of 250 mW/cm^2^. Honda et al. used N as a dopant and tetramethylsilane, 1,1,3,3-tetramethyldisilane, and n-hexane precursors. Demichelis et al. used SiH_4_ and CH_4_ gases, deposition temperature of 195 °C, and pressure of 640 mTorr. Donercark et al. used SiH_4_, CH_4_, and H_2_ gases at 200 °C and 1200 mTorr. Data from [57,58,79,115]. Reproduced with permission.

Three studies [58,79,115] have examined the variation of the optical bandgap with stoichiometry (Figure 6), finding a decrease in the bandgap as the Si content increases. Mastelaro et al. [116] attribute the previous trend to the formation of diamond-like C–C bonds at high C content rather than to voids due to hydrogen inclusion and the formation of microvoids. The high-C behavior of the bandgap is reported to depend on the bonding structure: graphite-like bonds cause a reduction in the optical bandgap for C concentrations above 50%, while C–C diamond-like bonds (achieved by silane-starving conditions) allow increases in the bandgap to continue for C concentrations up to 70% [105].

Honda et al. [57,117] observed a significant increase in the bandgap of N-doped C-rich a-SiC by decreasing the RF power during deposition including achieving optical bandgaps of 1.25 eV using 26 W RF power, opposite the expected trend from the change in composition observed in Section 3.1.2. Their deposition was optimized to reduce the formation of C–C and Si–Si clusters, which, combined with the presence of dopants, may explain the difference in their results.

In conclusion, the optical bandgap of a-SiC is reported to vary from 1.25 eV [117] to 4.0 eV [105], most strongly correlated to the composition, with higher C content being associated with a higher bandgap. The bandgap is reported to increase significantly by decreasing the RF power. Systematic studies on the effect of deposition temperature on the bandgap values are missing.

#### 3.3.2. Refractive Index

a-SiC’s wide bandgap makes it an almost ideal optoelectronic material, transparent for all visible wavelengths above 0.5 μm and thus highly suitable for guiding light in the visible and infrared optical spectrum. A refractive index typically greater than 2.5 (significantly larger than that of SiO_2_ and even than that of Si_3_N_4_) also makes α-SiC an excellent candidate for optical waveguides [74]. The refractive index has been used as a measure of the repeatability of a deposition recipe [6] and has been seen to be consistent across multiple depositions while using the same parameters. As a function of wavelength, the refractive index increases rapidly with the wavelength for small wavelengths and reaches a maximum near 400 nm and decreasing exponentially to a stable value in the near-IR range [16,66]. Refractive indices reported in the literature are typically measured at a wavelength between 500 nm and 677 nm.

**Figure 7:**
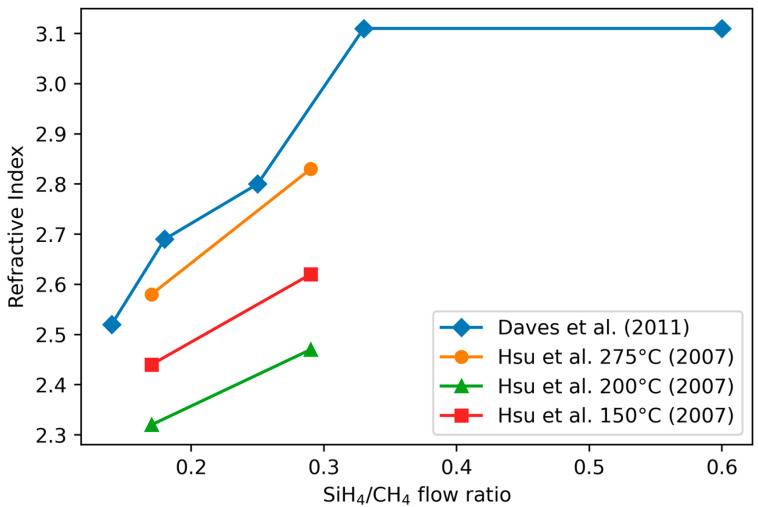
Variation of refractive index with SiH_4_/CH_4_ precursor flow ratio. Daves et al. used a temperature of 400 °C, 200 W RF power, and 1000 mTorr pressure, and measured the refractive index at 630 nm. Hsu et al. used three different temperatures, 300 W RF power, and 400 mTorr pressure, and measured the refractive index at 500 nm. Data from [19,55]. Reproduced with permission. Data from [60,82] are not available for reproduction.

Refractive index values reported in the literature are typically measured at a wavelength between 500 nm and 677 nm and they are between 1.66 and 3.4 [6,16,20,60,82,118]. The refractive index is mostly determined from the film Si and C composition [19,55,60,82], although the H content and porosity play a role [6]. The refractive index increases with increasing Si content according to all studies (Figure 7).

Increasing the hydrogen content decreases the refractive index for all Si/C compositions and temperatures studied [6,19]. This is associated with a decrease in film density and an increase in nanoporosity, which explain the changes in optical properties. Post-deposition annealing, which densifies the film and removes hydrogen, is found to moderately increase the refractive index, for example, an increase of 0.5 when annealed at 1000 °C [119].

No relation was observed between the refractive index and the RF power in [74], and there was only a small increase in the refractive index with increasing pressure (from 2.7 to 2.9 as the pressure increases from 500 mTorr to 2000 mTorr).

#### 3.3.3. Optical Properties of a-SiC Films for Biomedical Applications

Refractive index values for biomedical a-SiC are often not considered a critical parameter and thus are not always reported in the literature. Values of 1.7–2.4 are reported by Diaz-Botia [60], Hsu et al. [19] obtain values ranging from 2.3 to 2.6, and Chen et al. [16] obtain values from 1.93 to 2.53, which span most of the range obtained in a-SiC. Chen et al. note that higher refractive indices (2.5 and above) are preferred for waveguide applications, but the precise value used for devices is not mentioned in most biomedical studies. While a-SiC has seen relatively little use in biophotonic, optogenetic, or imaging applications, its transparency and the ability to widely vary the refractive index could allow for such applications in the future in conjunction with crystalline SiC [120,121].

### 3.4. Stress in a-SiC Films

Controlling stress in a-SiC:H films is one of the most significant challenges for PECVD deposition, as it depends on the film composition and bonding structure, the substrate, and the chamber conditions. El Khakani et al. propose a model for the stress in which the film stress is given by the bonding states, thermal stress from the substrate, and ion bombardment [69].

(1) $$\sigma_{total}=-AN_{C-H}-BN_{Si-H}+CN_{Si-C}+\sigma_{th}+\sigma_{b}$$

*A*, *B*, and *C* are experimentally determined positive constants; *N_C–H_*, *N_Si–H_*, and *N_Si–C_* are the bond densities of C–H, Si–H, and Si–C bonds, respectively. The *σ_th_* represents thermal stress from non-equal thermal expansion coefficients for the a-SiC film and the substrate, and *σ_b_* is the stress induced by ion bombardment during deposition. The—sign represents contributions to compressive stress.

Alternately, Daves et al. [55] propose that the stress occurs due to the coalescence of separately growing islands during deposition.

#### 3.4.1. Variation of Stress with Film Composition

**Figure 8:**
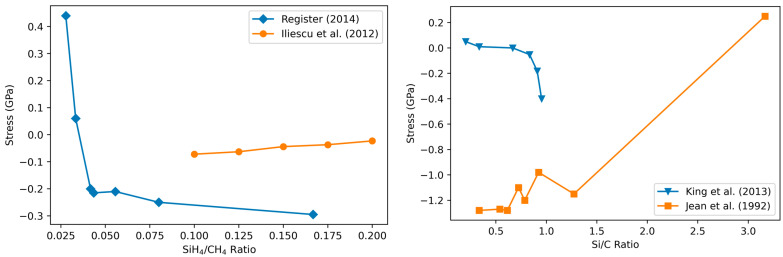
Stress as a function of stoichiometry or gas-flow composition. Both precursor gas ratio and measured composition are shown on separate axes. Compositional studies were performed using RBS (King et al.) and XPS/ERD (Jean et al.). Register used 250 °C, 50 W, and 900 mTorr, while Iliescu et al. used 300 °C, 150 W, and 1100 mTorr. King et al. varied most deposition parameters in order to obtain H-rich films. Jean et al. used a temperature of 250 °C and a pressure of 200 mTorr. Data from [6,20,93,122].

Several studies report the importance of the Si/C composition for the stress in a-SiC films (Figure 8). Studies in which the C content is increased while keeping all other parameters constant find an increase in the compressive stress [93,122], which matches the expected result from (1) given that H content tends to follow C content [95]. On the contrary, Register et al. [20] observed a consistent increase in tensile stress for increasing C content due to high contamination with oxygen during deposition that is not accounted for in (1).

Hydrogen content also influences the film stress as both Si–H and C–H bonds are expected to contribute to compressive stress according to model (1). The results of Windischmann et al. [123] and Herrera-Celis et al. [54] are in agreement with this model. Herrera-Celis et al. systematically study the bond densities of C–H, C–H_2_, and C–H_3_ as a function of the stress for a series of films, observing the expected trend of increasing compressive stress for increasing C–H bonding; however, they do not provide the stress values as a function of absolute H or C content.

King et al. [6] obtained a conflicting result, observing an increased tensile stress for increased C content and (correspondingly) H content (certified by the high porosity of their C- and H-rich films as well as by RBS measurements), in contradiction to the above results and model (1). The use of high-energy bombardment was proposed as a possible explanation for this behavior. Hsu et al. [19] also found that the compressive stress increases with decreasing hydrogen content, as certified by the corresponding decrease in Si–H and C–H bonds observed in the infrared spectra. The authors do not propose any physical explanation for these results.

In addition, Pham [80] found that the compressive stress is maximal (−600 GPa) for low SiH_4_ precursor flow (50 standard cubic centimeters per second, sccm), and becomes less compressive (−150 GPa) for an increased Si flow rate (250 sccm). If the C precursor is held constant, this result is in agreement with [93,122] and model (1) as described above. Other studies do not report a specific effect from the flow rate as opposed to the ratio of precursors.

In conclusion, while simple descriptive model (1) accurately predicts film stress variation with the film composition, changing multiple parameters simultaneously leads to significantly more complicated effects. 

#### 3.4.2. Variation of Stress with RF Power

**Figure 9:**
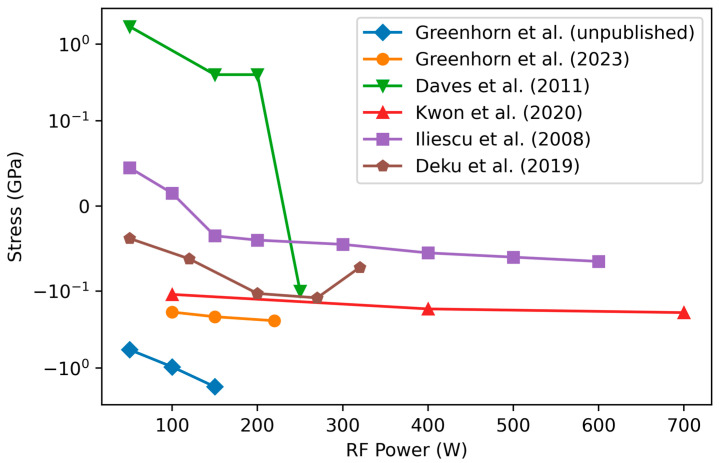
Residual stress as a function of plasma power. The studies by Greenhorn et al. use C_2_H_4_ as a C precursor, and the unpublished data use much lower precursor flow rates (23 sccm in total). Daves et al. used 1000 mTorr pressure and high temperature (400 °C) with a SiH_4_/CH_4_ ratio of 0.25, as well as a total flow rate of 200 sccm (90% Ar) while Kwon used a large industrial-type PECVD chamber with high flow rates, a pressure of 2500 mTorr, and a very high temperature of 550 °C and precursor gases of Si_2_H_6_ and CH_4_. Iliescu used a temperature of 300 °C, power of 150 W, and pressure of 1100 mTorr, and precursor/carrier flow rates of 70 sccm/500 sccm. Deku used a power density of 0.27 W:cm^2^ and a pressure of 1000 mTorr and fixed the total gas flow at 800 sccm. Data from [55,84,85,89,118]. Reproduced with permission.

Decreasing the RF power makes the residual stress more tensile according to [12,54,55,118] or has only a small influence on the stress [84,85,89] (Figure 9). Recall that decreasing RF power results in increased H content (see Section 3.1.1) while also decreasing the bombardment energy. Referring again to (1), this suggests that the contribution from the decreased bombardment energy outweighs or equilibrates that of the increase in hydrogen content. It is noted that in the Greenhorns’ unpublished data and the study of Daves et al. [55], low precursor flow rates are used, which may explain the significantly larger influence of the RF power.

#### 3.4.3. Variation of Stress with Deposition Pressure

Daves et al. [55] found a weak compressive stress increase with chamber pressure for pressures below 1500 mTorr. Above 1500 mTorr, a dramatic increase in compressive stress is observed. This is in agreement with Equation (1) as high pressure results in a decreased bombardment energy, σ_b_. Another study finds a nonlinear relation between stress and pressure, with a minimum compressive stress at 800 mTorr [54]. Pham, by contrast, finds that very high pressures (2500 mTorr as compared to 1500 mTorr) result in more tensile stress [80], contrary to model (1).

Note, however, that all studies observe compressive stress in all cases, indicating that the other deposition conditions favored compressive stress.

#### 3.4.4. Variation of Stress with Deposition Temperature

**Figure 10:**
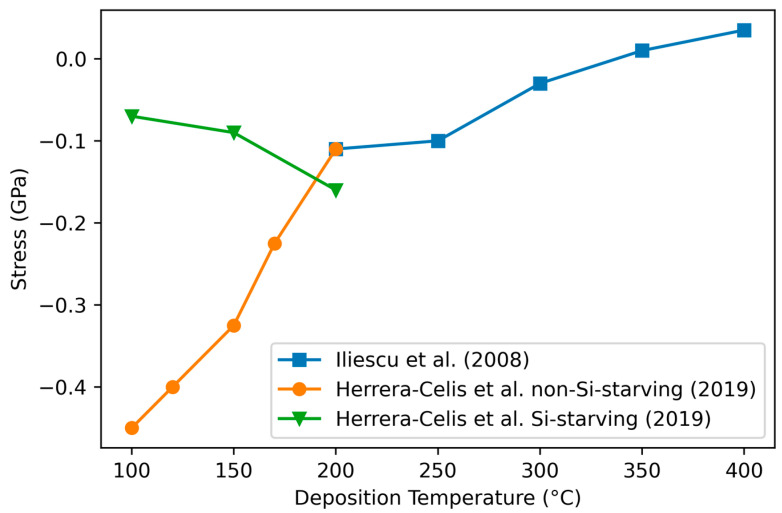
Iliescu et al. [118] show increasing residual stress with temperature, while Herrera-Celis [54] found two modes depending on the type of plasma: SiH_4_-starving and non-SiH_4_-starving. Iliescu et al. used a power of 150 W, pressure of 1100 mTorr, and reported films near stoichiometric composition. Herrera-Celis used power density of 16.7 mW/cm^2^ for the non-Si-starving case, 8.3 mW/cm^2^ for the Si-starving case, and pressure of 1100 Torr for both cases. The composition of the films was not reported in either study. Reproduced with permission.

According to Iliescu et al. [118], the deposition temperature has no effect on residual stress in the case of the low-frequency (380 kHz) generator while it has a dramatic effect in the case of the typical high-frequency (13.56 MHz) RF generator, with the stress becoming more tensile as the deposition temperature increases while the stoichiometry is not modified (Figure 10). Since an increase in temperature is expected to result in increased Si–C bonding (see Section 3.1.1), this result is consistent with the above relation, relation (1).

A more complex effect is observed by Herrera-Celis et al. [54], who found that the relationship between stress and temperature has two modes, depending on the species of radicals in the plasma chamber. In the “silane-starving regime” [116], the stress becomes more tensile as the temperature increases, in agreement with other studies. However, increasing the silane fraction (SiH_4_/(SiH_4_ + CH_4_)) to 0.26 inverses the trend, with the stress becoming increasingly compressive as the temperature increases in agreement with Hsu et al. [19] under similar conditions. This behavior agrees with (1) as, in the silane-starving regime, one expects a low probability of the reaction between silane radicals and methane, thus reducing the C–H bonds and increasing the formation of Si–C bonds.

#### 3.4.5. Modifying Film Stress with Post-Deposition Annealing

Post-PECVD-deposition annealing modifies the stress of the a-SiC layers [55], where the stress typically becomes more tensile after annealing due to the reduction in hydrogen content and C–H bonds. Typically, films with as-deposited compressive stress of several hundred MPa can be made to have tensile stress (of up to, again, several hundred MPa) after annealing in air at 600 °C [55,69,80]. The physics behind this effect was studied in detail by Frischmuth et al. [119] using mass effusion measurements, and they found that the change in stress is initially caused by the removal of H_2_, CH_4_, and Ar starting at 350 °C–400 °C and continuing to 900 °C, along with a corresponding decrease in film thickness and an increase in density. At 1000 °C, a second rapid increase in tensile stress occurs, also attributed to H_2_. Guivarc’h et al. [124] report similar results, with the evacuation of H beginning at 400 °C, and confirm the decrease in film thickness and density during annealing. 

King et al. [6] also notes a small increase of approx. 80 MPa toward tensile stress after heating up to 400 °C in a vacuum, a change which slowly reverses during exposure to air. It is suggested that this smaller effect is due to the evacuation of moisture during heating, which is then reincorporated after the experiment, returning the stress to a normal value. 

#### 3.4.6. Evolution of Film Stress over Time

Several studies [6,84,89,125] observe that films deposited at low temperature or at low RF power exhibit increased compressive stress over time. Samples maintained in an N_2_ atmosphere do not exhibit such a change. The change is attributed to the incorporation of O into the bulk of films by replacing Si–H bonds after deposition, which increase the stress by deforming the lattice structure. This conclusion is supported by XPS measurements showing an increase in oxygen in the material. The films deposited at low power have the lowest stress, but the stress increases significantly as a function of time, approaching that of films deposited at higher power. Another study notes that water vapor is particularly effective at oxidizing a-SiC [77].

Jousseaume et al. [125] reported decreasing the change in stress by using plasma treatments after deposition using He, O_2_, and H_2_. All treatments result in a more consistent and lower stress over time. Based on compositional measurements, different mechanisms are provided for each of the treatments. The He plasma increases the density of the film, the O_2_ plasma forms a dense oxide at the surface, and the H_2_ plasma passivates the dangling bonds. 

The contribution from incorporated oxygen to the stress is not included in Formula (1). While it is reported to be associated with the more porous, H/C-rich films, it suggests that the actual dynamic stress measured in a-SiC films exposed to typical environments is indeed more complicated than what (1) initially suggests.

In conclusion, a-SiC films deposited at both low RF power and low temperature (so, more porous structure with high H content) are the most vulnerable to a stress increase, likely due to oxygen passing through the porous structure [99]. An additional plasma treatment reduces the surface reactivity and improves the moisture barrier properties of the films, providing further evidence that the evolution in stress over time is caused by the incorporation of moisture.

#### 3.4.7. Stress in a-SiC Films for Biomedical Applications

Achieving minimal stress is a critical point for biomedical devices, with most studies achieving values below 200 MPa for their optimized films [12,18,20,39,60], compressive in all cases. High stress can lead to small cracks in the passivation [11] or, for thin implantable devices, an unwanted curvature that complicates insertion and placement [39]. Optimizing the stress has been one of the major focuses for biomedical a-SiC studies, and thus several different strategies for achieving low stress have been demonstrated. A careful balancing of the process parameters (deposition temperature, RF power, and pressure depending on the study) is necessary as there are multiple contributors to both tensile and compressive stress. Unfortunately, most studies do not account for changes in stress over time, which can vary significantly particularly in humid (biological) environments, which could significantly affect in vivo performance. The use of plasma post-treatment and/or thermal annealing has not been tested for biomedical applications, but has shown good results in minimizing changes in stress over time.

### 3.5. Mechanical Properties of a-SiC Films

a-SiC is noted for its desirable mechanical properties, in particular, a higher hardness/elastic modulus ratio than that of single-crystal SiC, suggesting its potential in wear-resistant applications [51], as well as extremely high tensile strength [126]. The maximum Young’s modulus is consistently achieved with 1:1 stoichiometric a-SiC films. While hardness is less studied, it typically follows the similar trends to the Young’s modulus.

#### 3.5.1. Variation of Film Mechanical Properties with Stoichiometry

**Figure 11:**
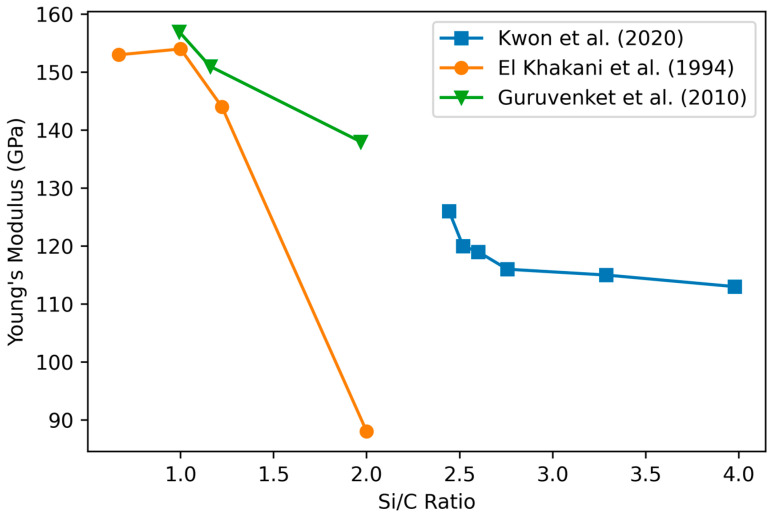
Comparison of results obtained by different studies comparing Young’s modulus to composition. Compositional studies were performed using XPS (Kwon et al.), ERD (El Khakani et al.), and ERD-ToF (Guruvenket et al.). Kwon et al. used a large industrial-type PECVD chamber with high flow rates, a pressure of 2500 mTorr, and a very high temperature of 550 °C and precursor gases of Si_2_H_6_ and CH_4_. Guruvenket et al. used temperature of 300 °C and pressure of 100 mTorr. El Khakani et al. used a temperature of 250 °C and pressure of 200 mTorr with a power density of 0.3 W/cm^2^. Data from [36,81,85]. Reproduced with permission.

Guruvenket et al. [81] found that the Young’s modulus and hardness decrease for increasing atomic Si/C ratios from 0.9 to 2, which correlate to a decrease in Si–C bonding (Figure 11). Janz et al. [79] also report increasing hardness with increasing C content for Si-rich films, in agreement with the other results (data not available for reproduction). On the contrary, Adithi et al. [82] determined that the Young’s modulus ranges from 124 to 167 GPa, roughly aligned to an increasing Si fraction, with Si/C ratios ranging from 0.85 to 1.2 (data not available for reproduction).

El Khakani et al. [36] studied hydrogenated (27%) and H-free SiC thin films fabricated with varying techniques including PECVD on undoped (100) Si substrates. They show that the nearly stoichiometric a-SiC:H films present higher hardness and Young’s modulus values than the Si-rich a-Si_x_C_1_*_−_*_x_:H films. Hydrogen-free a-SiC films present both hardness and Young’s modulus values (about 30 GPa and 240 GPa, respectively) higher by about 50% than those of hydrogenated a-SiC:H PECVD films. This study related the Young’s modulus linearly to the level of Si–C bonding in the material, finding maximal Young’s modulus with the maximal Si–C bonding at stoichiometric 1:1 Si:C. King et al.’s [6] results agreed with this conclusion.

Matsuda et al. [127] found that the hardness and Young’s modulus are inversely proportional to the porosity, which is associated with high C and H content. For increasing porosity/H content beyond a composition-dependent threshold, the films are found to become flexible. The importance of chemical ordering for the increased rigidity of Si-rich films is also emphasized in [109].

A study of a-SiC film doped with N at up to 40% [100] found improvements in the scratch resistance, nanoindentation resistance, and fracture resistance. Several of the improved surface properties are attributed to the formation of a thin layer of SiO_x_ on the surface, which is softer than a-SiC and prevents crack formation on the surface. The use of an oxide layer to improve the mechanical properties of a-SiC has been further studied by Bae et al. [128] for a-SiC fibers with similar results.

#### 3.5.2. Variation of Mechanical Properties with Deposition Temperature

Ivashchenko et al. [73] also found that the hardness and Young’s modulus increase on average with increasing temperature, although not linearly. This is likely caused by the effusion of H molecules at higher temperatures, which also typically results in increased Si–C bonding, in alignment with the previous conclusions.

#### 3.5.3. Variation of Mechanical Properties with Post-Deposition Annealing

The hardness and Young’s modulus of films annealed at 600 °C or higher have been observed to increase compared to their as-deposited values [119]. The effect is most likely explained by the reformulation of Si–C bonds after the effusion of hydrogen from the film.

#### 3.5.4. Mechanical Properties of a-SiC Films for Biomedical Applications

Most of the effort for optimizing the mechanical properties of a-SiC has focused on non-biological applications, requiring dedicated microresonator fabrication that is not pursued by biomedical studies. As such, hardness and Young’s moduli are not reported in these studies. For biomedical implants, an important consideration is the tissue–implant interface and mechanical mismatch. Due to the high hardness and Young’s modulus of a-SiC, it is impossible to directly match the properties of soft tissue with a-SiC. However, a SiC probes can be thinner and more compliant/flexible than the current Si-based implantable devices, leading to a reduced biotic response. The idea of employing stiff but thin (<10 μm) a-SiC for a minimal biotic response was the basis of flexible MEAs’ development with excellent results from Deku et al. [39]. It is really the device stiffness, which includes cross-sectional area, rather than just the device modulus, that seems to matter the most for a reduced biotic response [129].

### 3.6. Passivation Properties

The following sections address additional aspects of a-SiC film performance specific to biomedical device applications. 

#### 3.6.1. Dissolution Rate in Chemical Solutions

The dissolution rate of a-SiC:H films in chemical solutions is film-thickness-dependent. Indeed, Diaz-Botia et al. [60] tested the dissolution rate of their films in PBS in the case of thin films (380 nm), and many rectangle defects appeared after two weeks while a 650-nm-thick film remained intact and defect-free after a 6-week soaking test. The etch rate of a 110-nm-thick film was 0.05–2 nm/h at 90 °C in PBS whilst a 450-nm-thick film had no measurable etching. Likewise, Cogan et al. [18] observed a dissolution rate of 0.1 nm/h at 90 °C in a PBS solution, and no appreciable dissolution at 37 °C after 40 weeks in PBS for 1-µm-thick films. So, thick a-SiC films > 1 µm have a higher dissolution resistance than thin ones.

The deposition temperature having a strong impact on the film organization also plays an important role in dissolution resistance. a-SiC layers deposited at lower than 200 °C suffered from large quantities of rectangle defects after a 1-week PBS soaking test [19]. More dissolution-resistant films were deposited at 200 °C in silane-starving conditions. These results suggest that a low Si–C bond density and porous film may provide little protection in a saline solution. Diaz-Botia [60] also found that films deposited at higher temperatures have a reduced long-term leakage current through pinholes under accelerated aging in PBS, as samples deposited at 200 °C show a dramatic increase in the leakage current (from ~0 to 1250 nA/cm^2^) after 18 h whereas samples deposited at 350 °C do not show such an increase even after 600 h. The effect is most strongly visible for thin films (180 nm), whereas films above 500 nm do not show early breakdown. Contrary to the above studies, high chemical resistance for low-temperature depositions has been achieved [130], using a deposition temperature of 180 °C as compared to the more typical 300 °C–500 °C. This was achieved using He as the carrier gas and by increasing the silane precursor flow, avoiding the porosity associated with higher C contents.

Chemical resistance tests using other chemicals like HF and KOH have also been performed [131], demonstrating low etch rates. Avram et al. [132] tested in 30% KOH at 80 degrees and observed etching at a rate of 13 A/min, and no significant etching (below 10 A/h) in 49% HF. A clear trend between residual stress and chemical resistance was confirmed in an acid resistance test by using 40% HF [75] with no appreciable etching after the test for the lowest tensile stress (+41 MPa). It is noted that such chemical treatments may affect other film properties beyond just the removal of material; Iliescu et al. [13] found that KOH-treated a-SiC films had a significant reduction in biocompatibility when used for cell culturing.

In conclusion, the general trends in terms of chemical resistance are (i) that porous H-rich films with low Si–C bonding are more vulnerable to chemical etching while films with high Si–C bonding and low H content exhibit higher chemical resistance, and (ii) that thick films are more chemically resistant.

#### 3.6.2. Conformal Coating and Uniformity 

a-SiC films deposited by PECVD are quite uniform [60,118] with minimal defects [74] according to optical inspection and impedance tests in liquid, and are therefore acceptable for conformal coatings. Pham reports pinholes in Si-rich a-SiC but not in C-rich films [80]. In the case of Utah or metal-wire electrode arrays of needles with heights of the order of 2 mm, a ratio of 5 [18,19] to 10 [75] has been found between the thickness at the base and the tip of the probe, with a significantly thicker film at the wider base compared to the thin tip of the probes. When coated on implantable metallic stents, small cracks (<1 µm) were visible at junctions in the structure, which were noted as regions of high stress [11]. After torquing tests, an a-SiC coating on a Ti implant (with 20 nm SiO_2_ as an interfacial adhesion layer) remained unchanged, suggesting good adhesion [15].

#### 3.6.3. Surface Roughness

While the influence of the deposition conditions on a-SiC film roughness is unclear, the dependence on thickness is strongly supported by all available studies. Indeed, from AFM and SEM measurements, Deku et al. [39] observed a roughness of 4 nm RMS on 2-μm-thick a-SiC films, while Register et al. [20] found a surface roughness of 0.55 nm on 230-nm-thick a-SiC films. Herrera-Celis et al. [54] also report a roughness below 1 nm for all sub-micron films in their study. It seems that for sub-micron films, a roughness below 1 nm is observed while the latter increases to some nm for film thickness higher than 1 μm. Other studies using thicker films also find higher roughness, ranging from 2.4 to 6.1 nm [83,133]. In particular, the addition of NH_2_ gas as a precursor is reported to significantly increase the roughness, from 4 nm to 12 nm [134]. A 200 nm coating of a-SiC on Ti implants with roughness of 9.8 nm was found to reduce the roughness to 8.3 nm [15].

Gelvez-Lizarazo et al. [12] studied the variation of surface roughness systematically for low RF powers and low deposition temperatures and were able to obtain significant variation in the roughness (although they do not note the film thickness used). They found that the root-mean-square (RMS) roughness increases from 1.69 nm to 5.12 nm as the RF power increases from 15 W to 30 W, and increases from 0.88 nm to 5.12 nm as the temperature increases from 100 °C to 200 °C. They relate the surface roughness to the size of the nuclei attached to the surface in the early stages of deposition. At low pressure, there is increased inelastic scattering, leading to an increased dissociation of the silane and methane precursors, and thus the deposited nuclei are smaller.

## 4. Perspectives

Interest in a-SiC is increasing with new techniques and applications frequently emerging. Newer deposition techniques, including ICP CVD, are showing promise with high reliability and uniformity [67], but lack the large body of literature to perform a reliable comparative study of film property optimization. While ICP CVD also allows the use of lower temperatures during depositions, it remains unclear whether similar trends to those of PECVD will occur.

Application areas for a-SiC are also expected to increase, with recent results in ultrahard materials [126], as a platform for functionalization in biosensors [135], and as a platform for photoelectrochemical CO_2_ conversion [136]. Furthermore, new characterization techniques for C-based materials and amorphous materials could open up new possibilities for optimization, such as thermal-optic effects studied using two-wave mixing [137], bandgap measurements using Kelvin force microscopy [138], structural determination using atomic resolution electron tomography [139], and material simulations using molecular dynamics [140,141]. As a-SiC becomes easier to be reliably produced with a wider variety of optimized properties, it will see increasing use in novel devices.

## 5. Conclusions

Passivation with high conformability, biocompatibility, long lifetimes, high resistance, and low stress is critical for developing next-generation biomedical devices. While there has been significant work to optimize the deposition parameters and control the properties of PECVD a-SiC films, work remains to reliably obtain films with simultaneously optimal electrical resistivity, stress, and chemical resistance. Many properties of a-SiC film deposition are well understood and strongly supported by the literature, while others require additional study. With the innate high resistivity and excellent chemical resistance, a-SiC is a promising material for neural interface passivation if these properties can be preserved alongside low stress and desirable mechanical properties.

The most common parameter for tuning the film properties is the Si and C composition, which is primarily determined by the precursor flow rates of the Si- and C-containing species. Increasing the C content in a-SiC:H is known to increase the film electrical resistivity, decrease the refractive index, and increase the optical bandgap (and vice versa for increasing Si). Moving away from a 1:1 stoichiometry is also expected to decrease the hardness and Young’s modulus.

However, the Si and C composition alone does not fix all of the relevant properties. Another significant factor is the bonding and organization of the film, which is largely determined by H content. The hydrogen content can be increased by using a more C-rich precursor mixture, by decreasing the RF power, or by lowering the temperature. The resulting films have lower organization and are nanoporous, with low refractive indices and high resistivity due to the passivation of dangling bonds with H, and are mechanically more flexible, and tend to have low compressive stress. Their properties are also reported to be less stable in time, showing increased vulnerability to a chemical attack and increased oxygen intake when exposed to air, resulting in an increase in compressive stress. Alternately, using high RF power, high deposition temperature, or H_2_ flow during deposition has been shown to decrease H content and correspondingly increase Si–C bonding. These films are reported to be more stable with excellent chemical resistance and more stable properties over time.

While low-stress films are frequently reported, the effect of this optimization on other film properties, such as resistivity or chemical resistance, is not often considered. Stress control is one of the most important issues in PECVD a-SiC, with a wide range of stresses achievable from tensile to compressive. The most well-understood method to control the film stress is through the bonding structure, where Si–H and C–H bonds contribute to compressive stress and Si–C bonds contribute to tensile stress. However, ion bombardment during deposition can also lead to an increase in tensile stress that outweighs the contribution from bonding and is relatively understudied. Additionally, thermal mismatch with the substrate can contribute another source of stress, which must be considered when designing a recipe. Compressive stress can be reduced or made tensile by post-deposition annealing, at the expense of a decrease in resistivity.

The typical strategy for obtaining reliable a-SiC films for biomedical devices is to target a 1:1 stoichiometry using “silane-starving flow”, which results in dense, diamond-like films with high Si–C ordering. The addition of H_2_ gas flow and the use of high deposition temperatures further reduce disorder from terminating H bonds and increase Si–C bonding. Choices of RF power and gas flow rates will depend on the size of the reactor being used in order to achieve silane-starving flow. Note, however, that very low precursor gas flow rates (below 25 sccm) have resulted in films with high stress even in typical research-scale PECVD reactors.

It is clear that PECVD of a-SiC films is complicated to optimize given the significant interactions between process parameters that can influence all of the properties of the films. Moving forward, it is critical for the a-SiC research community to document all aspects of their processes and report a broad variety of characterization results in order to gain a more holistic understanding of the film growth.

## Figures and Tables

**Table 1:** Comparison of PECVD deposition systems using SiH_4_ and CH_4_ precursors for the studies of Figure 1.

Study	Daves [55]	Janz [79]	Pham [80]	Adithi [82]	Li/Guruvenket [81,83]
PECVD system	Oxford Plasmalab 100 Abingdon, UK	Roth & Rau AK400 Hohenstein-Ernstthal, Germany	Novellus Concept One San Jose, California	Oxford Plasmalab 100 Abingdon, UK	Applied Materials P5000 Santa Clara, CA, USA
Max Diameter (cm)	20	15.6	50	20	20
Power (W)	200	150	100	40	

## Data Availability

Restrictions apply to the availability of these data. Data were obtained from the respective copyright holders and are available in full from the original publications with the permission of the copyright holders.
